# Towards Accurate Biocompatibility: Rethinking Cytotoxicity Evaluation for Biodegradable Magnesium Alloys in Biomedical Applications

**DOI:** 10.3390/jfb15120382

**Published:** 2024-12-18

**Authors:** Angela De Luca, Roberta Ruggiero, Aurora Cordaro, Benedetta Marrelli, Lavinia Raimondi, Viviana Costa, Daniele Bellavia, Elisabetta Aiello, Matteo Pavarini, Antonio Piccininni, Marco Tatullo, Elisa Boanini, Francesco Paduano, Gianluca Giavaresi

**Affiliations:** 1 CS-Surgical Sciences and Technologies-SS Omics Science Platform for Personalized Orthopedics, IRCCS Istituto Ortopedico Rizzoli, 40136 Bologna, Italy; angela.deluca@ior.it (A.D.L.); auroracordaro97@gmail.com (A.C.); lavinia.raimondi@ior.it (L.R.); viviana.costa@ior.it (V.C.); daniele.bellavia@ior.it (D.B.); gianluca.giavaresi@ior.it (G.G.); 2 Stem Cells and Medical Genetics Units, Biomedical Section, Tecnologica Research Institute and Marrelli Health, 88900 Crotone, Italy; roberta.ruggiero@calabrodental.it (R.R.); benedetta.marrelli@calabrodental.it (B.M.); elisabetta.aiello@tecnologicasrl.com (E.A.); 3 Department of Chemistry, Materials and Chemical Engineering ‘G. Natta’, Politecnico di Milano, 20135 Milan, Italy; matteo.pavarini@polimi.it; 4 Department of Mechanics, Mathematics and Management, Polytechnic University of Bari, Via Orabona 4, 70125 Bari, Italy; antonio.piccininni@poliba.it; 5 Department of Translational Biomedicine and Neuroscience, DiBraiN, University of Bari “Aldo Moro”, 74124 Bari, Italy; marco.tatullo@uniba.it; 6 Department of Chemistry “Giacomo Ciamician”, Alma Mater Studiorum, University of Bologna, 40126 Bologna, Italy; elisa.boanini@unibo.it

**Keywords:** magnesium alloys, cytotoxicity, biodegradable alloy, extraction methods

## Abstract

Magnesium and its alloys represent promising candidates for biomedical implants due to their biodegradability and mechanical properties, which are similar to natural bone. However, their rapid degradation process characterized by dynamic pH fluctuations and significant hydrogen gas evolution during biocorrosion adversely affects both in vitro and in vivo assessments. While the ISO 10993-5 and 12 standards provide guidelines for evaluating the in vitro biocompatibility of biodegradable materials, they also introduce testing variability conditions that yield inconsistent results. To address these inherent characteristics of Mg alloys, developing improved methods that accurately simulate the physiological environment for in vitro biocompatibility testing is essential. This study introduces two novel extraction approaches for evaluating Mg alloys: a buffered solution utilizing PBS/DMEM with quaternary dilutions and a modified ISO standard protocol employing decuple dilution of conventional unbuffered extracts. The present findings establish that controlled optimization of extraction conditions, specifically buffer composition and dilution parameters, enables reliable in vitro cytotoxicity assessment of Mg alloys, providing a robust methodology that advances the preclinical evaluation of these promising biodegradable materials.

## 1. Introduction

Recent scientific research has increasingly focused on biodegradable metallic materials, such as magnesium (Mg), iron (Fe), and zinc (Zn), particularly within the context of biomedical applications [1]. These biodegradable metal matrices present promising alternatives, addressing the limitations of permanent bio-inert metal-based devices, as substantiated by numerous studies [2,3,4,5]. Conventional metallic substrates like stainless steel, titanium, and cobalt chrome alloys have been favored for their excellent mechanical properties, including high tensile strength, ductility, and corrosion resistance [6]. However, several clinical studies have indicated that metal ions or wear particulates from these durable metals can induce inflammatory responses in the surrounding biological environment, potentially causing aseptic loosening, osteolysis, and eventual implant failure, thus necessitating revision surgery [7,8,9].

Biodegradable materials, such as Mg and its alloys, represent promising candidates for implants, providing stability during healing and controlled degradation post-healing, thereby eliminating the requirement of secondary surgery and reducing risks of chronic inflammation or allergic response [10,11]. Mg alloys are particularly suitable for biomedical applications due to their bone-regeneration capabilities and mechanical properties analogous to natural bone [12]. As a crucial mineral, Mg is involved in various physiological processes, with about 50% residing in the skeletal system’s bone matrix [13]. Unlike bioinert alloys, biodegradable Mg alloys significantly alter both the local microenvironment and the alloy itself [14]. While Mg is biocompatible, its rapid corrosion in chloride-rich bodily fluids can harm surrounding tissues [15]. The inconsistent corrosion rates of Mg-based materials present challenges for implants, necessitating new alloy compositions and processing techniques for enhanced stability [16]. Although many studies explore developing Mg alloys with specific chemical components or coatings to attenuate degradation, they often overlook technical challenges [17]. Notably, in vitro tests frequently reveal significant alterations in the culture conditions due to the degradation of the Mg-based substrate [18]. Most in vitro setups are closed, static systems that hinder degradation product flow, complicating the study of Mg and its alloys, particularly in direct cell contact. These systems do not adequately replicate the dynamic environment of degradable implants in the body. An initial step to address these challenges involves conducting indirect contact tests using extracts. However, generating these extracts often results in elevated osmolality and pH, leading to in vitro cellular death from osmotic shock [19].

The ISO 10993:12 standard [20], which addresses the preparation of extracts for various biocompatibility assessments outlined in ISO 10993:1 [21] does not provide specific guidelines for degradable materials, such as Mg and its alloys [22]. For instance, when assessing the cytotoxicity of Mg alloys through indirect methods involving the utilization of alloy extracts, it is pivotal to note that the specific conditions for extract preparation remain notably indistinct within the existing literature [1]. Moreover, prevailing in vitro test systems cannot replicate the intricate homeostatic mechanisms that govern the modified microenvironment through physiological interactions, rendering it challenging to readily embrace the outcomes derived from prevailing ISO standard assessments [20,23]. Ethical considerations and the imperative for rapid in vitro pre-screening necessitate the establishment and optimization of robust test systems [24].

This study was designed to investigate the effects of different extraction vehicle formulations on the proliferation of various cell cultures, with particular emphasis on Mg alloy AZ31 extracts, thereby providing comprehensive insights into the optimal formulation that ensures robust in vitro results. The research methodology adheres to ISO 10993-5 (Tests for in vitro cytotoxicity) and 12 (Sample preparation and reference materials) standards, incorporating strategic modifications to extract preparation from different cell media [20]. To more accurately simulate the in vivo environment, we implemented improved extract preparation methods based on ISO 10993 parts 5 and 12, utilizing serum-supplemented cell culture media [25] and systematically controlled extract dilution [1,24,26]. Cytotoxicity tests were conducted using three distinct cell lines, mouse fibroblast L929, human osteosarcoma MG63, and mouse osteoblastic progenitor MC3T3-E1 cell lines, in strict accordance with ISO 10993-5 [23].

The present study aspired to illuminate the central role of extraction vehicle composition in cellular experiments involving Mg-based devices and provide a suggestion for future research in the field of cytotoxicity assessment. As numerous in vitro studies have potentially overestimated the cytotoxicity of Mg-based devices due to the closed environment inherent to in vitro conditions, this study aimed to identify more favorable in vitro conditions for establishing cytotoxicity levels of Mg-based devices.

## 2. Materials and Methods

### 2.1. Mg Alloy AZ31 Sample Preparation

The AZ31 magnesium alloy utilized in this investigation was sourced from a commercial supplier (Magnesium Elektron, Madison, IL, USA), with a manufacturer-certified composition containing 2.5–3.5% Al, 0.7–1.3% Zn, 0.2–1.0% Mn, 0–0.04% Ca, 0–0.05% Cu, 0–0.005% Fe, 0–0.05% Si, and 0–0.005% Ni, with the remainder being Mg. AZ31 magnesium alloy was selected due to its established commercial standardization and extensively documented biocompatibility profile in biomedical applications, with controlled aluminum content (2.5–3.5 wt%) demonstrating acceptable physiological tolerance. A 1 mm thick AZ31 disk (initial diameter equal to 75 mm) was initially deformed via the Superplastic Forming (SPF) process into a benchmark part characterized by a uniformly thinned hexagonal flat region, as described in a previous study [27]. The process was carried out using a laboratory 30 ton hydraulic press machine [28], and the blank was deformed under the action of a properly modulated flow of pressurized Ar gas (which was necessary to deform the material under an ideal regime of strain rate) after reaching the ideal temperature of 450 °C [29]. From the flat portion of the formed part, disks that were 10 mm in diameter were extracted via laser cutting. After production, SPF AZ31 samples were degreased by washing them in acetone (24201, Merck, Germany) for 5 min in an ultrasonic bath (Elmasonic S60, Elma Ultrasonic, Singen, Germany). To achieve surfaces free of contaminants and inhomogeneous oxides while keeping the mass loss of each sample below 5%, the specimens were acid pickled through immersion in an optimized solution [30] composed of 4 M acetic acid (695092, Sigma-Aldrich, Riedel-de Haën, Germany) and 1 M nitric acid (07006, Riedel-de Haën, Sigma-Aldrich, Germany) for 10 s, thoroughly rinsed in Milli-Q water with ultrasound for 5 min, and then rapidly dried with compressed air. The implants were individually placed in PA/PE double pouches, thermo-sealed, and then gamma-sterilized at 25.0 kGy.

### 2.2. Extract Preparation

The vehicle used to prepare the extracts was different for each method.

Method 1: The extraction vehicle was formulated by combining phosphate-buffered saline (PBS) devoid of Ca and Mg (Euroclone S.p.A., Milan, Italy) with a high-glucose DMEM cell culture medium (Euroclone S.p.A) in various proportions: 100% PBS, 75% PBS with 25% DMEM, 50% PBS with 50% DMEM, 25% PBS with 75% DMEM, and 100% DMEM. Each extraction vehicle was placed in contact through immersion with Mg AZ31 disks at a ratio of 0.2 g per ml volume. This setup was incubated at 37 °C in a 5% CO_2_ atmosphere for 72 h under static conditions. Post-incubation, the supernatant was carefully collected and subjected to centrifugation to remove particulate matter. The resulting extracts were then stored at 4 °C for up to three days before use. The pH of each extract was measured using a CyberScan pH 1100/2100 m (Eutech Instruments Pte Ltd., Blk 55, Ayer Rajah Crescent, Singapore), revealing an alkaline pH value of approximately 8. The influence of the magnesium extracts was assessed through cytotoxicity testing on L929 cell cultures. These tests involved applying each undiluted Mg extract (100%) as well as diluting them with Dulbecco’s minimum essential medium (DMEM) to achieve varying concentrations of 75%, 50%, 25%, and 10%.

Method 2: The Mg AZ31 extract was prepared by diluting the extraction vehicle DMEM tenfold, following the methodology outlined by Fischer et al., and supplemented with 10% fetal bovine serum (FBS) (S.I.A.L group, Rome, Italy) [22]. To obtain the desired concentration, the DMEM containing 10% FBS was added to the samples, maintaining a sample weight to extraction volume ratio of 0.2 g per 10 mL. The mixture was then incubated at 37 °C in a 5% CO_2_ atmosphere for 72 h.

### 2.3. Cell Culture

The in vitro assay was performed to evaluate the behavior of different cell cultures exposed to two different Mg extraction media. The following cell lines were used for the viability assay: murine fibroblast L929 (ATCC, LGC Standards S.r.L, Milan, Italy) and osteosarcoma cell line Mg-63 (ATCC, LGC Standards S.r.L, Milan, Italy). They were cultured in DMEM, supplemented with 10% fetal bovine serum (FBS), 100 U/mL of penicillin, 100 mg/mL of streptomycin, and 2mML-glutamine (Euroclone S.p.A., Milan, Italy) in standard conditions (37° C, 5% CO_2_, humidified atmosphere). The murine calvarial osteoprogenitor cell line MC3T3-E1 (ATCC, LGC Standards S.r.L, Milan, Italy) was cultured in Alpha Minimum Essential Medium (αMEM) (Gibco, Thermo Fisher Scientific Inc., Waltham, MA, USA), which lacks ascorbic acid but contains ribonucleosides, deoxyribonucleosides, 2 mM L-glutamine, and 1 mM sodium pyruvate, with 10% FBS. Incubation was performed at 37 °C in a 5% CO_2_ humidified atmosphere. After incubation, cells were detached using 0.1% trypsin–EDTA solution (Euroclone S.p.A., Milan, Italy) centrifuged at 1200 rpm for 5 min and resuspended in cell culture medium.

### 2.4. Cell Culture Treatment and WST-1 Analysis for Determination of Correct Extraction Vehicle of Method 1

To evaluate which of the five Mg extracts prepared through Method 1 produced the best cellular response in terms of viability (reaching values closer to 80% than the control culture), L929 cell cultures were seeded at a density of 5 × 10^3^/96-well plates. The cells were then treated with 100 µL of Mg extracts (100% PBS, 75% PBS with 25% DMEM, 50% PBS with 50% DMEM, 25% PBS with 75% DMEM, or 100% DMEM) and incubated for 24 and 72 h at 37 °C in a humidified atmosphere containing 5% CO_2_.

### 2.5. Determination of Mg Content Through MP-AES Technique

The amount of Mg was determined through molecular plasma atomic emission spectroscopy (MP-AES) using the Agilent 4210 (Agilent, Santa Clara, CA, USA). The Mg line at 518.36 nm was used. Analyses were performed through comparison with five calibration standards (0.5, 1, 5, 10, 20 mg/L) prepared through dilution of 1000 mg/L Mg standard solution in 0.5 M HNO_3_ (Sigma Aldrich, Merk Life Science S.r.l., Milan, Italy). Two extracts obtained through Method 1 and Method 2 were subjected to different dilutions to enter the linearity range of the calibration line. In particular, the extracts obtained through Method 1 were diluted 50-fold, and extracts obtained through Method 2 were diluted 5-fold. All of the data presented take into account the dilution factor for direct comparison. The results of this analysis are the average of three different determinations on two different replicates.

### 2.6. Cell Culture Treatment and WST-1 Analysis for Determination of Adequate Dilution of Extracts of Method 1

Subsequently, L929 cell cultures were seeded at a density of 5×10³ per 96-well plate and exposed to a series of dilutions of Mg extraction vehicle, formulated by combining 50% PBS with 50% DMEM, at concentrations ranging from 100% to 10%. For the WST-1 assay, 1/10 volume of WST-1 reagent was added to each well, and the cells were incubated at 37 °C in a 5% CO_2_ humidified atmosphere for 3 h. The absorbance of the samples was measured against the background (culture medium plus WST-1 reagent without cells) at 450 nm using a BioTek 800 TS microplate reader (BioTek Instruments, Winooski, VT, USA). Absorbance values obtained from the WST-1 assay are proportional to the total number of viable cells. Subsequently, the combination selected as the Mg extraction vehicle (50% PBS + 50% DMEM) was diluted to 25% in the culture medium of all cell lines analyzed at 24 and 72 h.

### 2.7. Method 1 and Method 2 Comparative Analyses of Cell Viability

To assess the impact of selected Mg extracts prepared through Methods 1 and 2, the three cell lines were seeded at a density of 2.5 × 10^3^ with 100 μL/well Mg extract (50% PBS + 50% DMEM) at 25% of the total culture medium for Method 1 or with 100 μL/well Mg extracts at 100% of the total culture medium for Method 2. The cells were then incubated at 37 °C in a 5% CO_2_ humidified atmosphere for 24 and 72 h. Two viability assays, WST-1 (as described before) and PrestoBlue (Thermo Fisher Scientific Inc., Waltham, MA, USA) were performed on all cell lines in 96-well plates according to the manufacturer’s recommendations. The PrestoBlue assay was also used for viability analysis on the same cell lines after 24 and 72 h. Specifically, a 1X solution of PrestoBlue was added at a concentration of 100 μL/well and incubated for 4 h. Absorbance was measured at two different wavelengths, 570 nm and 600 nm, using a Multiskan GO microplate spectrophotometer (Thermo Fisher Scientific Inc., MA, USA). DMEM medium alone (100 μL/well) was used as a negative control in both assays.

### 2.8. Statistical Analysis

The statistical analysis was conducted utilizing the StataNow 18.5 software (StataCorp. 2023. Stata Statistical Software: Release 18.5. StataCorp LLC., College Station, TX, USA). Following the verification of data distribution and homogeneity via the Shapiro–Wilk and Levene tests, respectively, repeated-measure data (n = 3 biological replicates for n = 3 technical replicates) were examined using a multilevel mixed-effects linear regression model. Subsequently, the significance of multiple comparisons was adjusted using the Sidak method.

## 3. Results

### Evaluation of the Effect of Mg Extracts from Method 1 and Method 2 on Cell Culture Proliferation

**Figure 1:**
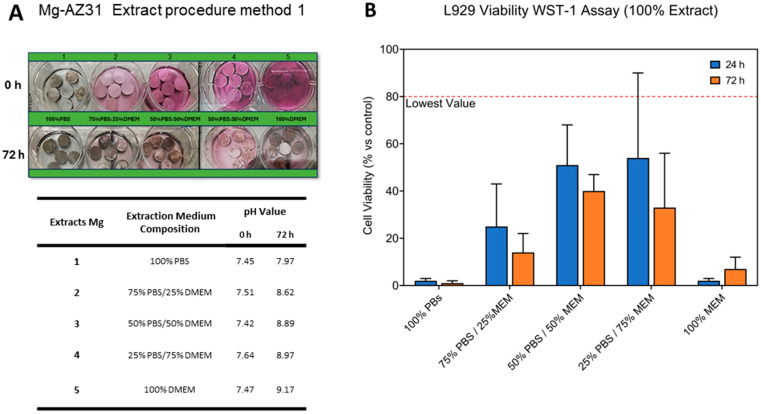
(**A**) Representative image of extract preparation through Method 1. The table shows the pH level at 0 and 72 h for each extract prepared using Method 1. (**B**) WST-1 viability assay on L929 cell lines to evaluate the effect of several compositions (PBS: DMEM) of the extraction vehicle through the Method 1 protocol (Mean ± SD, n = 4).

To evaluate the effects of the extraction vehicle used in extraction Method 1 on cell viability, a selection of PBS formulations was systematically developed in combination with DMEM cell culture medium. The formulations were prepared in specific proportions to evaluate their respective efficacy in cell viability. Specifically, the formulations for Method 1 extraction included a vehicle composed entirely of PBS (100%), a mixture of 75% PBS combined with 25% DMEM, a balanced formulation of PBS and DMEM (50% PBS + 50% DMEM), a variant with 25% PBS and 75% DMEM, and a vehicle formulated with DMEM alone (100%) (Figure 1A). The table presented in Figure 1A shows the pH variation of the different prepared extraction vehicles and demonstrates how the presence of PBS solution reduces the basicity of the medium.

These extracts were incubated with the cells and provided the basis for the subsequent cell viability experiments. The results of the cell viability WST-1 assay on the L929 cell line were expressed as a percentage compared to the control culture, which was maintained in culture without Mg extract. These results showed that the best cell viability at 24 and 72 h was obtained with the 50% PBS and 50% DMEM extraction solution compared to the other formulations (Figure 1B).

**Figure 2:**
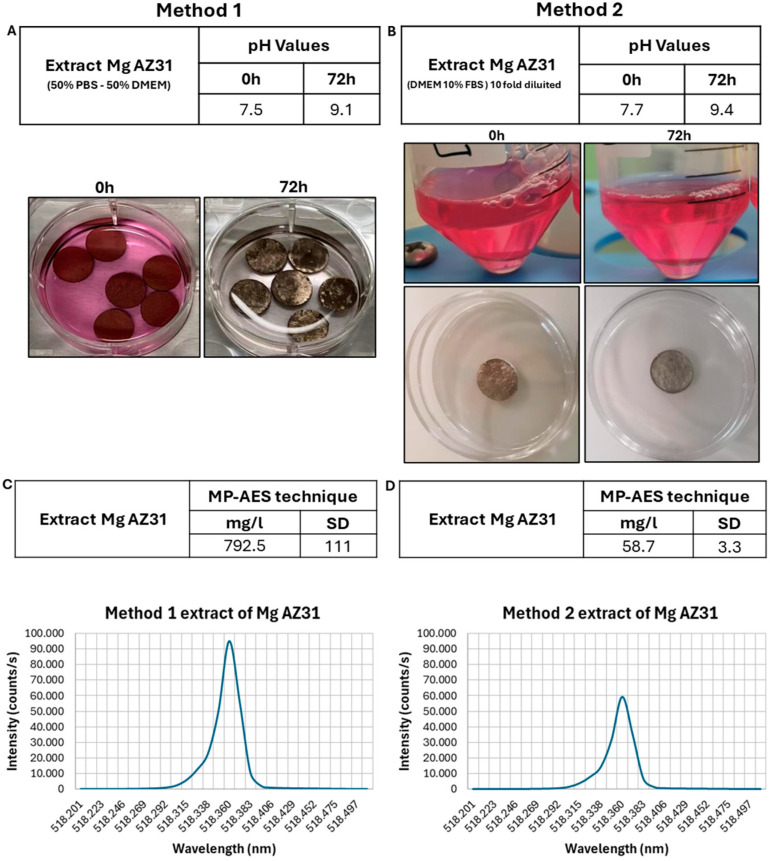
(**A**,**B**) Representative image of Mg AZ31 samples during extract preparation. The table shows the pH value at 0 and 72 h for each extract prepared through Methods 1 (**A**) and 2 (**B**). (**C**,**D**) Each graph shows the concentration (intensity) of the Mg element (518.360 nm Mg line reference) in each extract analyzed. Analysis results are the average of three different determinations on two different replicates.

After identifying the most efficient extraction vehicle for Method 1, characterization of Mg content was conducted using the MP-AES technique for both Method 1 and a modified ISO10993-12 protocol (Method 2, detailed in the subsequent section) [20]. Initial pH measurements of extracts were recorded, followed by quantitative determination of Mg concentrations (Figure 2A,B). Baseline Mg content in the extraction vehicles utilized in Method 1 resulted in 2.73 ± 0.05 mg/L, while in Method 2 it accounted for 11.74 ± 0.47 mg/L. Following incubation with Mg AZ31, the Mg concentration in the extract of Method 1 was found to be 792.5 ± 111.0 mg/L, while that of Method 2 was 58.70 ± 2.35 mg/L. Figure 2C,D show the representative MP-AES spectral profiles, reflecting method-dependent variations in Mg quantification due to the distinct extraction vehicles employed.

**Figure 3:**
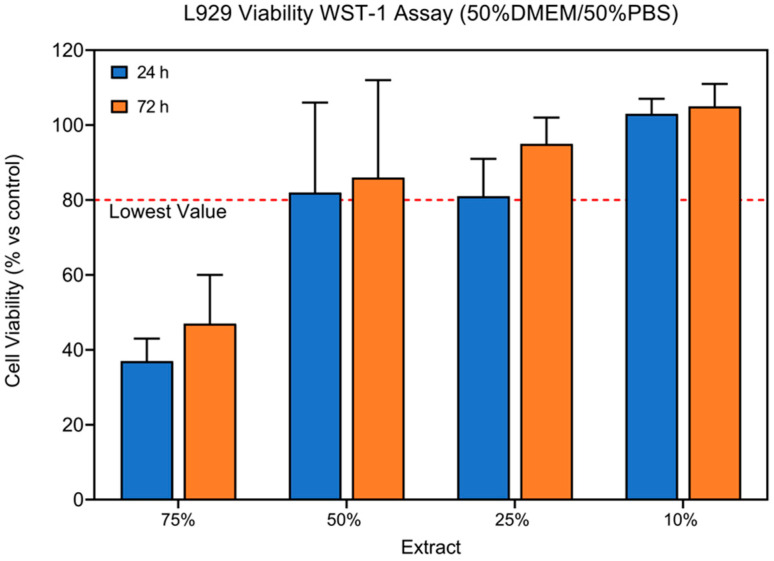
WST-1 viability assay on L929 cell lines to evaluate the effect of several dilutions of 50%PBS:50%DMEM extract vehicle (Mean ± SD, n = 4).

Subsequently, additional WST-1 analysis was conducted on L929 utilizing varying concentrations of 50% PBS with 50% DMEM extract. The cell viability results indicated that the 25% dilution of the 50% PBS with 50% DMEM extract ensured a minimum viability of at least 80%, which is the safety level according to ISO 10993-5, with a coefficient of variation of 0.74–0.12 (24 h: 81 ± 10%; 72 h: 95 ± 7%) (Figure 3). The data enabled the identification of the optimal extraction vehicle, comprising 50% PBS and 50% DMEM, with an appropriate dilution of 25%.

After the determination of optimal parameters for Method 1, an alternative extraction method (Method 2) based on a modified ISO 10993-12:2012 protocol was then investigated [20]. Although ISO guidelines specify a standardized sample-to-vehicle ratio of 0.2 g/mL (1X concentration), initial evaluation of magnesium substrates under these conditions exhibited substantial cytotoxic effects. We therefore developed a modified protocol implementing a tenfold dilution strategy in DMEM (10X) of the standard 1X extracts, prepared according to ISO 10993:12 parameters [20], based on the methodological approach pioneered by Fischer et al. [25]. Quantitative assessment of cellular metabolic activity demonstrated that L929 fibroblasts exposed to the decuple-diluted extract (Method 2) exhibited robust viability exceeding the critical 80% threshold at both 24 and 72 h post-exposure, validating both the biocompatibility of the Mg alloy and the reliability of this modified testing approach (Supplementary Figure S1).

**Figure 4:**
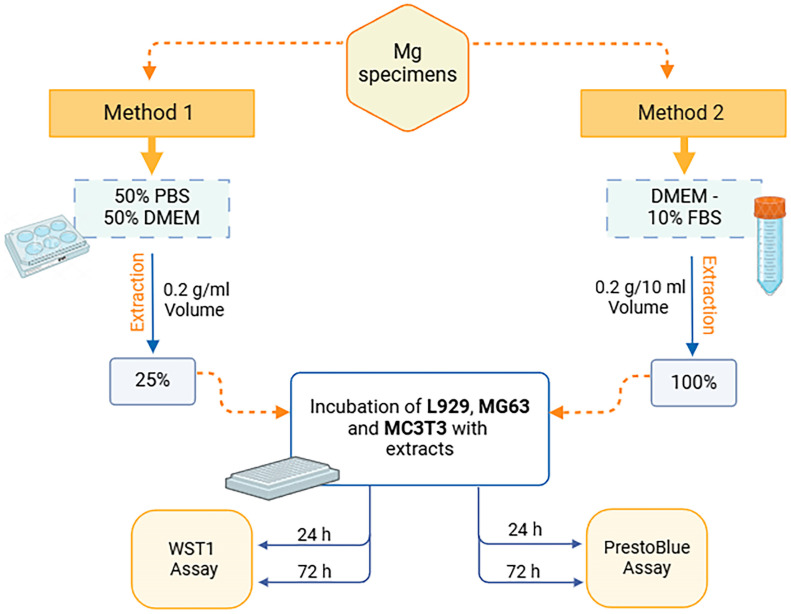
Schematic overview of two extraction methods (Method 1 and Method 2) evaluated using WST-1 and PrestoBlue viability assays in three different cell lines.

To further validate these findings and establish a comprehensive assessment of the extraction methodologies, we conducted parallel analyses using both WST-1 and PrestoBlue viability assays across multiple cell lineages (Figure 4). These complementary metabolic assays were strategically employed to evaluate the comparative effects of both extraction Methods 1 and 2 on L929 fibroblasts, MG63 osteosarcoma cells, and MC3T3 osteoblastic progenitors, representing diverse tissue-specific responses relevant to biomedical applications.

**Figure 5:**
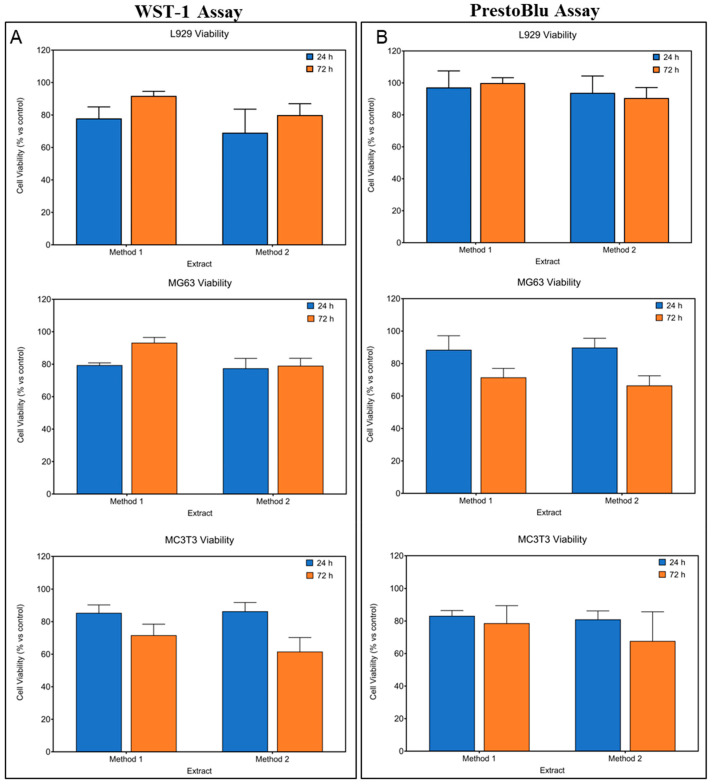
Comparative analysis of the effect of two extraction methods on the viability of different cell lines. (**A**) WST-1 cell viability assay performed on L929, MG63, and MC3T3 cell lines; (**B**) PrestoBlue cell viability assay performed on L929, MG63, and MC3T3 cell lines. Statistical analysis revealed no significant differences in cell viability between the two extraction methods and only a significant reduction over time in MC3T3 measured with WST-1 (*diff* = −19.23, *F* = 22.56, *p* < 0.0005) and in MG63 with PrestoBlue (*diff* = −20.18, *F* = 60.94, *p* < 0.0005).

The results demonstrated that extraction Method 1, utilizing the optimized 25% dilution protocol, and Method 2, employing the modified ISO protocol with decuple dilution, both maintained cellular viability above 80% across all tested lineages (Figure 5A,B). Statistical analysis revealed no significant differences in cell viability between the two extraction methods. Both the PBS/DMEM buffered system with quaternary dilution (Method 1) and the modified ISO standard protocol with decuple dilution (Method 2) were validated as reliable protocols for assessing Mg alloy biocompatibility. These findings demonstrate that controlled optimization of extraction conditions, such as the buffered PBS/DMEM system with 25% dilution or the tenfold-diluted ISO standard protocol, enables reproducible in vitro cytotoxicity assessment of Mg alloys.

## 4. Discussion

A careful analysis of the literature reveals a significant disparity between the biocompatibility assessment outcomes of Mg-based medical devices under in vitro and in vivo conditions [31]. Several scientific articles evaluating the biosafety of different Mg devices have applied extraction methods and cytotoxicity tests that deviate from the ISO standards [24,32]. These standards specify procedures for the biological evaluation of implant materials and outline key requirements, including (1) experimental protocols for indirect cytotoxicity testing; (2) cell type selection; (3) a medical device extract preparation protocol; and (4) cell–extract contact periods [33].

The in vivo system is dynamic and can induce changes in the environment of a degradable material [34]. Indeed, human blood consists of cells, plasma containing proteins, and corrosive ions, such as Cl^−^, which form a complex electrolyte for Mg alloys, such as Mg chloride (MgCl_2_), which is water-soluble and separates into Mg^2+^ ions and chlorine ions [35]. The Mg^2+^ ions produced by Mg alloy degradation in the human body are non-toxic and do not cause systemic toxicity, but they can promote new bone formation, accelerating the bone healing process. This allows for the use of Mg alloys as biofunctional bone implants for orthopedics [36]. Therefore, ensuring in vitro extract preparation under conditions that more closely mimic physiological circumstances becomes imperative to accurately emulate expected in vivo conditions [37].

In this study, while the initial experimental methodology of the extraction and viability assessment strictly adhered to ISO standards, the indirect viability method unexpectedly revealed marked cytotoxicity in Mg substrates. These observations prompted us to develop alternative protocols for both extracts and vehicle compositions to achieve the 80% viability threshold recommended. Through systematic investigations, Method 1 demonstrated the crucial role of extraction vehicle composition in modulating L929 viability. Notably, an optimized extraction vehicle comprising an equal part of PBS and DMEM (50:50) significantly enhanced L929 cell viability to approximately 50%.

While this improvement was significant, we sought to further validate our findings through an alternative approach. Building upon the previous study, Method 2 adopted the strategy of Fischer et al., wherein the extraction vehicle underwent tenfold dilution with respect to the ISO 19993-12 standard weight/volume ratio [25]. This complementary methodology provided an important comparative framework for evaluating extraction protocols.

To evaluate these distinct approaches, the quantitative analysis through MP-AES analysis unequivocally demonstrated that the amount of Mg present in both extracts obtained through Method 1 and 2 is markedly disparate, with Method 1’s extract being approximately 13.5 times more concentrated than that of Method 2. Despite these concentration differences, cells in contact with extract 1 diluted in 25% cell culture medium achieved cell viability of 80%, aligning with the guideline requirements. Importantly, these viability data were replicated by testing 25% of extract 1, and no significant statistical differences were found when comparing the effects of extraction methods on all cell lines tested using two different viability tests.

The results showed that reducing the amount of Mg in the extract and diluting both extracts (Method 1 by a factor of 4 and Method 2 by a factor of 10) yields almost identical viability data. As documented in the Hassan et al. study, chemical degradation reactions between the AZ31 Mg disk and the DMEM solution result in the release of hydroxyl ions (OH^−^) into the environment, leading to a rapid increase in pH and a reduction in cell viability [38]. Our results suggest that cells cultured in vitro (in static environment) with the extracts obtained through the two different methods can metabolize the OH^−^ ions produced over time within a certain range of Mg ion concentration. This can be defined as 50–200 µg/mL Mg, within which reliable viability data can be obtained. Despite the higher levels of Mg reported in Method 1, the mixed composition of the extracting vehicle in the presence of PBS helps to reduce the release of OH^−^ ions, leading to better control of the pH increase, reflecting better cell viability, as demonstrated by Silva et al. [39].

On the other side, the observed lower concentration of Mg in extraction Method 2 is probably due to the presence of FBS in the extraction vehicle. Specifically, negatively charged proteins can inhibit Mg alloy degradation through competition with Cl^−^ in the aqueous environment. This reduces the concentration of Mg^2+^ ions in the extraction vehicle but does not completely stop the degradation of the surface alloy [40]. This could be additional information to evaluate by including the presence of FBS in extract 1.

This study highlights the need to configure new experimental approaches to make in vitro testing of biodegradable materials reliable, repeatable, and comparable between laboratories. It demonstrates the importance of considering the effects of the preparation of material extracts on the in vitro cytotoxicity of biodegradable materials, which change dynamically with the environment, to obtain an accurate and valid assessment. Furthermore, the data obtained indicate that the ISO guidelines should be implemented by providing more specific suggestions on the preparation of extracts for different types of biodegradable materials.

In this study, by employing AZ31 magnesium alloy as a reference substrate, we establish two novel methodological approaches for quantitative cytotoxicity evaluation of magnesium-based biomaterials, demonstrating enhanced analytical capabilities compared to conventional ISO protocols. We postulate that these optimized methods exhibit potential applicability across diverse magnesium-based biomaterial systems beyond AZ31. This hypothesis is supported by systematic quantitative analyses across multiple AZ31 variants, including SPIF-manufactured specimens and surface-functionalized configurations incorporating β-TCP matrices [41,42,43]. However, the intrinsic compositional variations of magnesium-based alloys could necessitate comprehensive validation protocols specifically addressing both temporal degradation kinetics and constituent element-specific cytotoxicity profiles across diverse alloy compositions.

In this context, it is important to underline that the evaluation of magnesium-based biomaterials also requires consideration of factors beyond the extraction methodology. The observed ionic concentrations and resultant pH variations are governed not only by dilution parameters but also by the intrinsic corrosion kinetics of the alloy system. In AZ31 matrices, metallic impurities, predominantly iron and nickel species, constitute significant determinants of the degradation dynamics, introducing an additional dimension to the standardization of biocompatibility assessments.

Achieving robust translation between in vitro experimentation and in vivo outcomes necessitates the development of new experimental approaches that precisely define the optimal physiological parameters for cell cellular viability assessment, thus addressing the expanding complexity of biomedical engineering innovations. Although this study establishes standardized extraction protocols for cytotoxicity assessment, these methodologies warrant further validation through immunological response analyses and long-term tissue response. Integration of these biological parameters into standardized testing frameworks will be fundamental for advancing the clinical translation of magnesium-based biomaterials, ultimately bridging the gap between preclinical evaluation and therapeutic application.

## 5. Conclusions

Current ISO protocols for in vitro biocompatibility assessment require strategic refinement when evaluating biodegradable magnesium alloys. Systematic optimization of extraction parameters, specifically through controlled buffer composition and quaternary/decuple dilution strategies, established a reproducible cytotoxicity testing methodology.

The validated protocols effectively mitigated assessment challenges inherent to magnesium degradation, including pH fluctuation and hydrogen evolution, while maintaining cellular viability above the ISO-recommended 80% threshold. This research provides standardized cytotoxicity testing protocols for magnesium alloys, thus advancing the preclinical evaluation of these promising biodegradable implant materials.

## Data Availability

The original contributions presented in the study are included in the article/Supplementary Material, further inquiries can be directed to the corresponding author.

## Supplementary Materials

The following supporting information can be downloaded at https://www.mdpi.com/article/10.3390/jfb15120382/s1, Figure S1: PrestoBlue assessment of L929 fibroblasts exposed to decuple-diluted magnesium extracts (10X, Method 2).
