# Supplementary material for: Towards Accurate Biocompatibility: Rethinking Cytotoxicity Evaluation for Biodegradable Magnesium Alloys in Biomedical Applications
Source: J Funct Biomater. 2024 Dec 18;15(12):382. doi: 10.3390/jfb15120382 (PMC11678253; doi:10.3390/jfb15120382)

Article

Towards Accurate Biocompatibility: Rethinking Cytotoxicity Evaluation for Biodegradable Magnesium Alloys in Biomedical Application

De Luca et al.,

Supplementary Figure S1. PrestoBlue assessment of L929 fibroblasts exposed to decuple-diluted magnesium extracts (10X, Method 2).

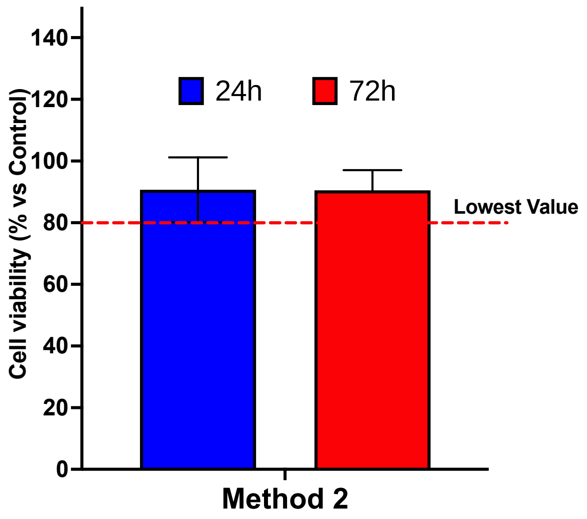

Supplement: Supplementary file 1 [file jfb-15-00382-s001.zip › jfb-3369973-supplementary.pdf]
